# Reprogramming lipid metabolism as potential strategy for hematological malignancy therapy

**DOI:** 10.3389/fonc.2022.987499

**Published:** 2022-08-29

**Authors:** Leqiang Zhang, Ning Chang, Jia Liu, Zhuojun Liu, Yajin Wu, Linlin Sui, Wei Chen

**Affiliations:** ^1^ School of Engineering Medicine, Beihang University, Beijing, China; ^2^ School of Biological Science and Medical Engineering, Beihang University, Beijing, China; ^3^ Beijing Advanced Innovation Center for Biomedical Engineering, Beihang University, Beijing, China; ^4^ Peking University Cancer Hospital, Beijing, China; ^5^ Core Lab Glycobiol & Glycoengn, College of Basic Medical Sciences, Dalian Medical University, Dalian, China

**Keywords:** lipid metabolism reprogramming, cholesterol, fatty acids, phospholipids, hematological malignancies

## Abstract

Hematological malignancies are one of the most lethal illnesses that seriously threaten human life and health. Lipids are important constituents of various biological membranes and substances for energy storage and cell signaling. Furthermore, lipids are critical in the normal physiological activities of cells. In the process of the lethal transformation of hematological malignancies, lipid metabolism reprogramming meets the material and energy requirements of rapidly proliferating and dividing tumor cells. A large number of studies have shown that dysregulated lipid metabolism, commonly occurs in hematological malignancies, mediating the proliferation, growth, migration, invasion, apoptosis, drug resistance and immune escape of tumor cells. Targeting the lipid metabolism pathway of hematological malignancies has become an effective therapeutic approach. This article reviews the oncogenic mechanisms of lipid metabolism reprogramming in hematological malignancies, including fatty acid, cholesterol and phospholipid metabolism, thereby offering an insight into targeting lipid metabolism in the treatment of hematological malignancies.

## Introduction

Hematological malignancies are a collection of malignant tumors that aberrant hematological cells or immune cells are blocked in differentiation and proliferate indefinitely, leading to the dysfunction of biological organisms (1). There are three main types of hematological malignancies: leukemia, multiple myeloma (MM), and lymphoma (2). Hematological malignancies are one of the most lethal illnesses that seriously threaten human life and health with a high mortality rate. According to the World Cancer Report 2020 released by the World Health Organization, the new number of non-Hodgkin’s lymphoma, leukemia, MM and Hodgkin’s lymphoma in 2020 were 544,352, 474,519, 176,404 and 83,087, respectively, accounting for 6.6% of the total number of patients. The corresponding number of deaths were 259,793, 311,594, 117,077 and 23,376 respectively, accounting for 7.1% of the total number of patients (3). Due to its particularity, hematological malignancies cannot be surgically removed like solid tumors, and its clinical first-line treatment options mainly include chemotherapy, radiotherapy and hematopoietic stem cell transplantation (4). Although the traditional first-line therapies have a certain effect, the overall efficacy is not optimistic due to the relapse/refractory caused by the occurrence of primary/secondary drug resistance (5). With the deepening of research, new cancer therapies have brought dawn to relapsed/refractory patients, including CAR-T cell therapies, ADC drugs and immune checkpoint inhibitors (6). The U.S. Food and Drug Administration approved anti-CD19 CAR-T cell therapy Tisagenlecleucel for the treatment of B-cell acute lymphoblastic leukemia (ALL) (7), ADC drug Loncastuximab Tesirine-Lpyl for the treatment of relapsed/refractory diffuse large B-cell lymphoma (DLBCL) (8) and PD-1 inhibitor Pembrolizumab for the treatment of Hodgkin lymphoma (9). However, even under novel therapies, there are still a large number of patients with poor clinical prognosis. Therefore, it is still a work with clinical application value and important scientific significance to study the oncogenic mechanism and find new therapeutic targets in order to develop new therapeutic methods (10).

It has been widely reported that cell metabolism affects tumor cell proliferation, apoptosis, migration, invasion, chemical resistance and immune escape (11–14). There is a close relationship between cellular metabolism and functional output, once the metabolic pathway is abnormal, leading to abnormal cell function and disease progression (15). Compared with normal cells, tumor cells undergo metabolic reprogramming due to their excessive proliferation, growth, migration and metastasis requiring faster and more energy, and tumor cell metabolic reprogramming has been identified as a new marker of cancer (16). Clinical observations found that lipid metabolism reprogramming often predicts poorer prognosis in cancer patients (17). A large number of lipid droplets that store lipids and cholesterol can be detected in tumor cells, high lipid droplets and high cholesterol esters are also considered indicators of cancer aggressiveness in tumor cells (18). *De novo* lipid synthesis pathway and uptake of exogenous lipids are often enhanced in rapidly dividing and energy-consuming tumor cells, such as malignant plasma cells from obese myeloma patients with high expression of acetyl-CoA synthase 2 (19). Acetyl-CoA synthase 2 is a key precursor for the *de novo* synthesis of fatty acids (FAs). Fatty acid synthase (FASN) is also upregulated in various hematological malignancies (20). The fatty acid transporter protein (FATP), which mediates cellular uptake of FAs, is expressed at high levels on both the cell surface and the intracellular space of patients with MM (21). Lipids are not only important components of organelles and energy substances, but also signaling molecules that are crucial for maintaining cellular homeostasis (22). Lymphoma-derived exosomes promote tumorigenesis by increasing lipid metabolism in recipient cells through surface phospholipase A2 (23). Lysophosphatidic acid (LPA)-mediated activation of the MEK1/2-ERK1/2 signaling pathway increases oxidative phosphorylation in the mitochondria of MM cells, which in turn produces large amounts of NAD^+^ and ATP. It impairs the activity of proteasome inhibitors and enhances protein folding in the endoplasmic reticulum (ER), thereby conferring resistance to proteasome inhibitors in MM (24). In this review, we discuss the lipid metabolism reprogramming and its oncogenic mechanisms in hematological malignancies, including FA metabolism, cholesterol metabolism, phospholipid metabolism and lipid-related signaling pathways.

## FA metabolism in hematological malignancies

**Figure 1:**
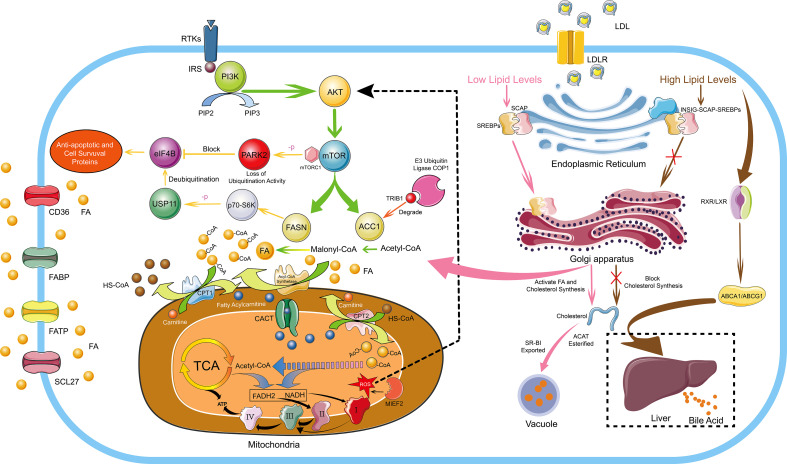
FA And Cholesterol Metabolism in Hematological Malignancies. The *de novo* synthesis of FA is regulated by the PI3K/Akt signaling pathway. *De novo* FA synthesis mainly depends on two key rate-limiting enzymes, ACC carboxylates acetyl-CoA to malonyl-CoA, and FASN converts acetyl-CoA and malonyl-CoA Conversion of acyl-CoA to long-chain FA. ACC and FASN are regulated by mTOR, which is a downstream target of the PI3K/Akt signaling pathway. Overexpressed FASN can prompt p70-S6 kinase to phosphorylate USP11 and the phosphorylated USP11 mediates eIF4B deubiquitination to increase its stability. eIF4B mediates the expression of anti-apoptotic and cell survival proteins, ultimately driving the development of lymphoma. In addition, PARK2 can also be phosphorylated by mTORC1 to lose its ubiquitination activity, thereby blocking the ubiquitination and degradation of eIF4B protein. The E3 ubiquitin ligase COP1 binds to ACC1 through Trib1 and causes ACC1 ubiquitination and degradation to inhibit FA synthesis. Cells uptake exogenous FAs mainly through CD36, FATP, FABP and SCL27. Endogenous FAs and exogenous FAs enter the mitochondria through the transmembrane mechanism, and generate a large amount of ATP through FA β-oxidation, the TCA cycle and the electron transport chains. Moreover, MIEF2, a key regulator of mitochondrial fission, can stimulate the production of mitochondrial ROS and activate the AKT/mTOR signaling pathway to further enhance FA synthesis. Cholesterol homeostasis is mainly regulated by SREBPs and LXRs. In conditions of low cholesterol levels, SCAP-SREBP2 can be smoothly transferred from the ER to the Golgi apparatus to activate the *de novo* synthesis pathway of cholesterol. In conditions of high cholesterol levels, INSIG, SCAP and SREBP2 form a stable trimolecular complex to block the export and activation of SREBP2, and finally inhibiting the *de novo* cholesterol synthetic route. Moreover, by activating LXR-RXR to express ABCA1 and ABCG1, excessive cholesterol is transported to the liver and excreted in the form of bile acids. Furthermore, excessive cholesterol is rapidly esterified and exported under the action of ACAT and SR-BI to form vacuoles containing cholesterol ester derivatives. I, II, III and IV represent complex I, complex II, complex III and complex IV in the electron transport chains, respectively.

FAs are essential molecules in the entire lipid metabolism, not only involved in the synthesis of biological membranes and secondary signaling molecules, but also substrates for mitochondrial ATP and NADH synthesis, eicosanoid production and post-translational protein–lipid modifications of signaling proteins (25, 26). As early as 1924, Warburg proposed that even under sufficient oxygen conditions, tumor cells also prefer the low-utilization form of glycolysis for energy production (27). Even if tumor cells use a large amount of carbohydrates, it is challenging to meet the needs of energy substances, so lipid metabolism is also required for energy. Tumor cells increase lipid metabolism and energy supply mainly by enhancing the *de novo* synthesis pathway of endogenous FAs, exogenous FAs uptake and lipid mobilization (28). *De novo* FA synthesis mainly depends on two key rate-limiting enzymes, acetyl-CoA carboxylase (ACC) carboxylates acetyl-CoA to malonyl-CoA, and FASN converts acetyl-CoA and malonyl-CoA Conversion of acyl-CoA to long-chain FAs (**Figure 1**) (29). It has been reported that the FA *de novo* synthesis pathway is generally up-regulated in tumor cells, and FASN overexpression has been an independent prognostic marker for the aggressive clinical course of tumor cells (30). MYC^+^ BCL-2^+^ DLBCL with high expression of FASN has the characteristics of high invasion and poor prognosis (31). Up-regulated FASN can promote the growth, metastasis, invasion and anti-apoptosis of DLBCL through the pERK/BCL-2 signaling pathway, and FASN inhibition can cause cell growth arrest and apoptosis (32). Overexpressed FASN can also regulate the PI3K/Akt signaling pathway in DLBCL, prompting p70-S6 kinase to phosphorylate USP11. The phosphorylated USP11 mediates eIF4B deubiquitination to increase its stability, and eIF4B promotes key oncogenes biosynthesis, ultimately driving the development of lymphoma (33). PARK2 can also be phosphorylated by mTORC1 to lose its ubiquitination activity, thereby blocking the ubiquitination and degradation of eIF4B protein (**Figure 1**) (34). Meanwhile, up-regulation of FASN has been reported in acute myeloid leukemia (AML) (35), mantle cell lymphoma (MCL) (36), and MM (37).

There is a lack of reports about ACC1 in hematological malignancies, only one recent paper mentioned the function of ACC1 to suppress tumors. The E3 ubiquitin ligase COP1 binds to ACC1 through Trib1 and causes ACC1 ubiquitination and degradation to inactivate its biological activity, which leads metabolic reprogramming to support the energy requirements of leukemia progression. A general downregulation of ACC1 can be observed in AML. However, stabilizing the biological activity of ACC1 protein can increase intracellular ROS levels and NADPH consumption, thereby inhibiting leukemia progression, which may be caused by the material and energy competition conflict between ACC1-mediated FA synthesis and tumor cell proliferation (38). As an effective strategy for the treatment of patients with AML and endemic Burkitt lymphoma, the drug combination of bezafibrate and medroxyprogesterone acetate can effectively reduce the expression of FASN and stearoyl-CoA desaturase 1. However, ACC1, which is also a key enzyme in lipid synthesis, did not show a significant change in expression (39). The role of ACC1 remains unknown in hematological malignancies. Interestingly, ACC1 promotes tumor cell growth in some solid tumors. MIEF2, a key regulator of mitochondrial fission, stimulates the production of mitochondrial ROS and activates the AKT/mTOR signaling pathway. The result causes the upregulation of ACC1, FASN, SREBP1/2, SCD1, HMGCS1 and HMGCR to increase lipid synthesis, ultimately promoting the growth and metastasis of ovarian cancer cells (40). Overexpression of the long non-coding RNA (lncRNA, CTD-2245E15) in lung cancer can also regulate ACC1 and pyruvate carboxylase to promote lung cancer development (41).

Tumor cells enhance the uptake of exogenous FAs, mainly through the CD36, FATP, lipid chaperone FA binding protein (FABP) and solute carrier protein family 27 (SCL27). Up-regulation of CD36 has been reported in hematological malignancies such as AML (42), chronic lymphocytic leukemia (CLL) (43), MM (44), DLBCL (30), and MCL (45). Sudjit Luanpitpong (45) used Synchrotron-Based Fourier Transform Infrared Spectroscopy of Single Cells to detect significant increases in total lipids and lipid esters in MCL resistant to Bortezomib (BZ). BZ is a protease inhibitor that leads to accumulation of misfolded and unfolded proteins, mainly by inhibiting protein degradation, ultimately causing the ER stress response. Subsequent Oil Red O staining detection revealed significant lipid droplets accumulation in BZ-resistant MCL cells. Detection of lipid metabolism-related targets revealed increased expression of CD36 protein responsible for exogenous FAs uptake, and CD36 inhibited apoptosis in MCL, which was associated with BZ-resistance. It has been reported that BTK inhibitors can inhibit lipid droplet accumulation in MCL (46). Moreover, CD36 is associated with tumor invasion and metastasis and is a prognostic biomarker for various types of cancer (47–49). Apolipoprotein C2, which is highly expressed in AML, can interact with CD36 to activate LYN-ERK signaling and enhance the metabolic activity of leukemia cells (42). CD36 was also found to promote FAs uptake by activating STAT3 in CLL (43). Up-regulation of CD36 is also one of the reasons why tumor cells develop drug resistance (50). Exogenous interleukin 6 (IL-6) mediates the up-regulation of CD36 by activating STAT3, promoting the uptake of FAs and causing chemotherapy resistance (51). CD36 also induces lipid peroxidation and ferroptosis with concomitant reduction of cytotoxic cytokines and impaired antitumor capacity (52). In addition to the drug resistance of tumor cells caused by increased lipid synthesis, decreased lipid synthesis can also lead to drug resistance. BZ exerts its anti-tumor effect through ER stress caused by the protein accumulation (53), because the ER is the site of lipid synthesis, and BZ also causes lipid accumulation (45). In BZ-resistant MM, it was found that the expression of SREBP1 and its downstream target FA elongase ELOVL6 are reduced, resulting in inhibiting lipid synthesis, thereby reducing the accumulation on the ER and ultimately causing BZ-resistance (54). In addition to CD36 mediating cellular uptake of FAs, FATP also mediates cellular uptake of FAs. FATP is expressed at high levels on the cell surface and intracellular space in MM. Furthermore, MM cells can induce lipolysis of bone marrow adipocytes, and the decomposed free fatty acids (FFA) are taken up by adjacent MM cells through FATP (21). FABP and SCL27 were also observed to be up-regulated in tumor cells, increasing the uptake of exogenous FAs of tumor cells (55–58). Glycoprotein prostaglandin D2 synthase (PTGDS) has dual roles in prostaglandin metabolism and lipid transport. More interestingly, PTGDS exhibits different functions in different tumor cells. PTGDS promotes DLBCL progression by regulating tumor cell viability, proliferation, cell cycle, apoptosis and invasion through MYH9 stimulated Wnt-β-catenin-STAT3 signaling pathway (59). However, PTGDS showed antitumor effect in testicular cancer (60), gastric cancer (61) and breast cancer (62).

Fatty acid oxidation (FAO) provides energy mainly through FA β-oxidation. In order to successfully carry out FAO, FAs first need to enter the mitochondria. Firstly, long-chain FAs need to generate fatty acyl-CoA under the action of fatty acyl-CoA synthase. Fatty acyl-CoA is converted to fatty acylcarnitine under the action of CPT1, which is then transported into the mitochondrial matrix by carnitine/acylcarnitine translocase (CACT) on the inner mitochondrial membrane, and fatty acylcarnitine entering the mitochondrial matrix are reconverted to fatty acyl-CoA by CPT2. The fatty acyl-CoA that smoothly enters the mitochondria repeats the cycle of dehydrogenation, water addition, dehydrogenation, and thiolysis, and finally decomposes the fatty acyl-CoA into acetyl-CoA, accompanied by the generation of a large amount of NADH and FADH2. These substances eventually enter the TCA cycle and the electron transport chains to be oxidized to generate a large amount of ATP for cellular physiological activities (**Figure 1**) (28). FAO is dysregulated in a variety of malignancies, and it mediates tumor cell proliferation, survival, drug resistance, metastatic progression, immunosuppression and tumor-promoting microenvironment (63). As an enzyme involved in FAO, HADHB is commonly overexpressed in malignant lymphomas and is a poor prognosis predictor in DLBCL, and high expression of HADHB promotes the proliferation and growth of malignant lymphomas (64). HADHA, which forms a heterodimer together with HADHB, is also widely up-regulated in malignant lymphomas, and down-regulation of HADHA can cause G0/G1 cell cycle arrest (65). Acyl-CoA oxidase 1 (ACOX1), a key rate-limiting enzyme in FAO, is overexpressed in malignant lymphomas and confers resistance to the anthracycline antibiotic doxorubicin, mainly by reducing doxorubicin-induced activation of caspase-9 and caspase-3 and reduction of mitochondrial membrane potential. Simultaneously, ACOX1 can also destabilize the tumor suppressor gene family p73 protein and inhibit its expression (66).

Lipid metabolism can affect the immune system in tumor cells, causing immune evasion and promoting tumor growth. Natural killer (NK) cells play an important role in the prevention of hematological malignancies. However, FAs, both in lymphoma cells and in the tumor microenvironment, can reprogram lipid metabolism in NK cells and inhibit the production of cytokines such as IFN-γ, making NK cells lose their immune function to tumor cells (67). The prostaglandin PGD 2, a lipid compound of the eicosanoid family, is abundantly generated under the catalysis of cyclooxygenase overexpressed in tumor cells. PGD 2 can stimulate innate lymphocytes ILC2 to overexpress IL-5, which subsequently promotes the proliferation of Tregs cells. Tregs cells can be involved in immunosuppression, such as inhibition of T effector cell proliferation and production restriction inflammatory response factor IL-10, ultimately promoting the proliferation of hematological stem and progenitor cells (68). Tumor-associated macrophages (TAMs) up-regulate CD36 to uptake lipids resulting in lipid accumulation. Excess lipids provide a large amount of energy through FAO and lead to activation of STAT6, which is accompanied by TAMs differentiation and cancer promotion (69).

Since FA biosynthesis, uptake, and oxidation are significantly enhanced in various types of tumor cells, inhibiting FA mobilization has become a promising antitumor strategy in tumor cells. FASN, a key rate-limiting enzyme in the *de novo* synthesis pathway of endogenous FAs, is associated with multidrug resistance in tumor cells (70) and is an effective target for the treatment of malignant tumors. Clinically, glucocorticoids such as prednisone and dexamethasone can inhibit the expression of FASN and thereby inhibit the proliferation and growth of tumor cells (71). At the same time, the study found that ginger extract can inhibit the expression of FASN, and combined use with dexamethasone can enhance the drug sensitivity of ALL cells to dexamethasone (72). The combination of bezafibrate and medroxyprogesterone acetate (39), orlistat (73), N-phenylmaleimide (74) and methyl jasmonate (75) can all regulate the expression of FASN to inhibit the growth of tumor cells. In leukemia cells, FABP4 regulates DNMT1 expression through the IL-6/STAT3 axis and DNMT1 controls FABP4 through VEGF signaling, thereby forming a mutually reinforcing positive feedback regulation that ultimately promotes AML aggressiveness. The selective inhibitor BMS309403 can cause FABP4 dysfunction, which in turn promotes the downregulation of DNMT1. Subsequent induction of global DNA methylation and re-expression of tumor suppressor genes ultimately induce AML cell differentiation and inhibit AML progression (76).

## Cholesterol metabolism in hematological malignancies

Cholesterol is an important substance for cell function, and cholesterol homeostasis is essential for the normal physiological activities of the body (77). Cholesterol homeostasis is mainly regulated by two transcription factor families, sterol regulator element binding proteins (SREBPs) and liver X receptors (LXRs). SREBPs mediate lipid synthesis and LXRs mediate cholesterol transport. Andrea Brendolan has made a detailed summary of cholesterol homeostasis regulation. In brief, in conditions of low cholesterol levels, SREBP2 is escorted to the Golgi apparatus by SREBP cleavage activator protein (SCAP), where a series of biological reactions activate cholesterol synthesis, and LXRs are in an inhibited state at this time. In conditions of high cholesterol levels, INSIG, SCAP and SREBP2 form a stable trimolecular complex in the ER, thereby blocking the export and activation of SREBP2 and finally blocking the *de novo* cholesterol synthetic route. Moreover, excess oxysterols or desmosterol bind and activate the LXR/RXR heterodimer, which in turn activates specific LXR target genes, such as ATP-binding cassette transporters A1 and G1 (ABCA1 and ASCG1), allowing excess cholesterol to be transported to the liver and excreted as bile acids (**Figure 1**) (78).

The mechanisms that maintain cholesterol homeostasis are disrupted in tumor cells due to their addiction to cholesterol. It has been reported that a large amount of cholesterol is widely present in malignant tumor cells (79). In human hepatocellular carcinoma cells, the stability of the INSIG, SCAP, and SREBP2 trimolecular complex is destabilized by cascade phosphorylation of the AKT-PCK1-INSIG axis. SCAP-SREBP complex is translated to the Golgi apparatus to activate cholesterol synthesis and ultimately promotes the proliferation and growth of tumor cells (80). Up-regulation of LDLR, SREBP2 and nuclear PBR are detected in CLL, which also explains that hypocholesterolemia in lymphocytic leukemia patients is due to over-uptake of LDL particles from plasma by high LDLR expression (81). At the same time, it has also been found that tumor cells can secrete cytokines through autocrine and paracrine mechanisms to stimulate cellular uptake of LDL in AML (82). In T-cell ALL, the Wnt-β-catenin signaling pathway mediates the oncogenic synergy of Akt and Dlx5 by enhancing cholesterol synthesis (83). Yajie Shen (84) found that SOX9 was highly expressed in the GC-DLBCL with IGH-BCL2^+^ mutation. Through whole transcriptome analysis and chromatin immunoprecipitation sequencing, it was found that SOX9 could directly bind and transcriptionally activate DHCR24, which is a terminal enzyme in cholesterol biosynthesis that catalyzes the conversion of sterol to cholesterol. Using simvastatin to inhibit cholesterol synthesis, it can effectively inhibit the growth of DLBCL and the progression of lymphoma. Moreover, peroxisome proliferator-activated receptor (PPARδ) is co-expressed with cholesterol synthesis-related genes. The expression level of the key cholesterol synthesis enzyme HMGCR increases nearly 4-fold in malignant B cells with high PPARδ gene expression, and a significant increase in membranous cholesterol was also observed in malignant B cells, indicating changes in cell signal pathways (85). In promyelocytic leukemia (APL) driven by the PML-RAPα oncoprotein, it was found that PML-RAPα can reduce the expression of PPARγ. PML-RAPα and TRIB3 cooperate to destroy the PPARγ/RXR heterodimer to inhibit PPARγ activity, eventually causing abnormal blood lipids in APL (86). Although cholesterol is necessary for maintaining cell homeostasis and cancer cell proliferation, excess free cholesterol is harmful to cells (87). Therefore, cholesterol is rapidly esterified and exported under the action of acetyl-coenzyme A:cholesterol acetyltransferase (ACAT) and scavenger receptor class B member I (SR-BI) to form vacuoles containing cholesterol ester derivatives (**Figure 1**). A large number of vacuoles have been observed in highly aggressive lymphoma cells, which have been shown to contain lipids by Sudan black positive staining, and up-regulation of molecules related to cholesterol metabolism has also been detected (88).

Cholesterol metabolism reprogramming is also one of the reasons for the drug resistance of tumor cells. HMGCS1, a key enzyme in the mevalonate pathway for cholesterol synthesis, is overexpressed by the upstream regulator GATA1 in patients with relapsed/refractory AML. Activated HMGCS1 protects ER and mitochondria by upregulating the unfolded protein response (UPR) signaling pathway to avoid cell damage caused by RE stress and mitochondrial stress, ultimately endow tumor cells with drug resistance (89). In the tumor microenvironment of MM, there is a large number of oxidatively modified low-density lipoproteins (OxLDLs). These OxLDLs make proteasome inhibitors such as BZ lose their inhibitory and pro-apoptotic effects on the proteasome, and finally make MM patients acquire drug resistance (90). Chemotherapy generally causes drug resistance in tumor cells. After chemotherapy of AML, cellular cholesterol biosynthesis is significantly up-regulated and extracellular vesicles carrying a large number of cholesterol synthesis-related enzymes are secreted, which can be uptake by recipient cells to promote cholesterol synthesis and cell signal transduction, resulting in tumor formation (91).

Because cholesterol affects the occurrence and development of tumors, inhibition of cholesterol synthesis has become a new strategy for the treatment of cancer in recent years (92). In DLBCL, the BCR/SYK/PI3K/AKT signaling pathway regulates the biosynthesis of cholesterol (93). Metformin, a commonly used drug for the treatment of diabetes, can reduce the biosynthesis of cholesterol by blocking the BCR signaling pathway and inhibiting the expression of HMGCS1, thereby inhibiting the growth of DLBCL. Furthermore, limiting the biosynthesis of cholesterol affects the integrity and biological function of the cell membrane and the lipid rafts, further blocking the BCR signaling pathway and the cell activity is severely inhibited (94). Nicotinamide phosphoribosyltransferase inhibitor (KPT-9274) can selectively kill leukemia stem cells (LSC) and is an effective treatment for AML. However, due to the up-regulation of SREBP-regulated genes, LSC developed a certain resistance to KPT-9274 (95). Inhibiting the SREBP signaling pathway with dipyridamole can enhance the drug sensitivity of LSC cells to KPT-9274. In addition, simvastatin, a common statin that reduces plasma cholesterol levels, has been reported to promote apoptosis by inhibiting the miR-19a-3p/HIF-1α axis (96). Conversely, increasing cholesterol biosynthesis can also kill tumor cells. Overactivation of ERK/MAPK signaling pathway using limonoid compounds A1542 and A1543 induces excessive cholesterol biosynthesis in leukemia cells, leading to cholesterol accumulation and programmed apoptosis in leukemia cells (97). In addition, blocking cholesterol efflux by SR-BI inhibitor, resulting in intracellular cholesterol accumulation, can also stimulate ER stress response and eventually lead to apoptosis (88).

## Phospholipid metabolism in hematological malignancies

**Figure 2:**
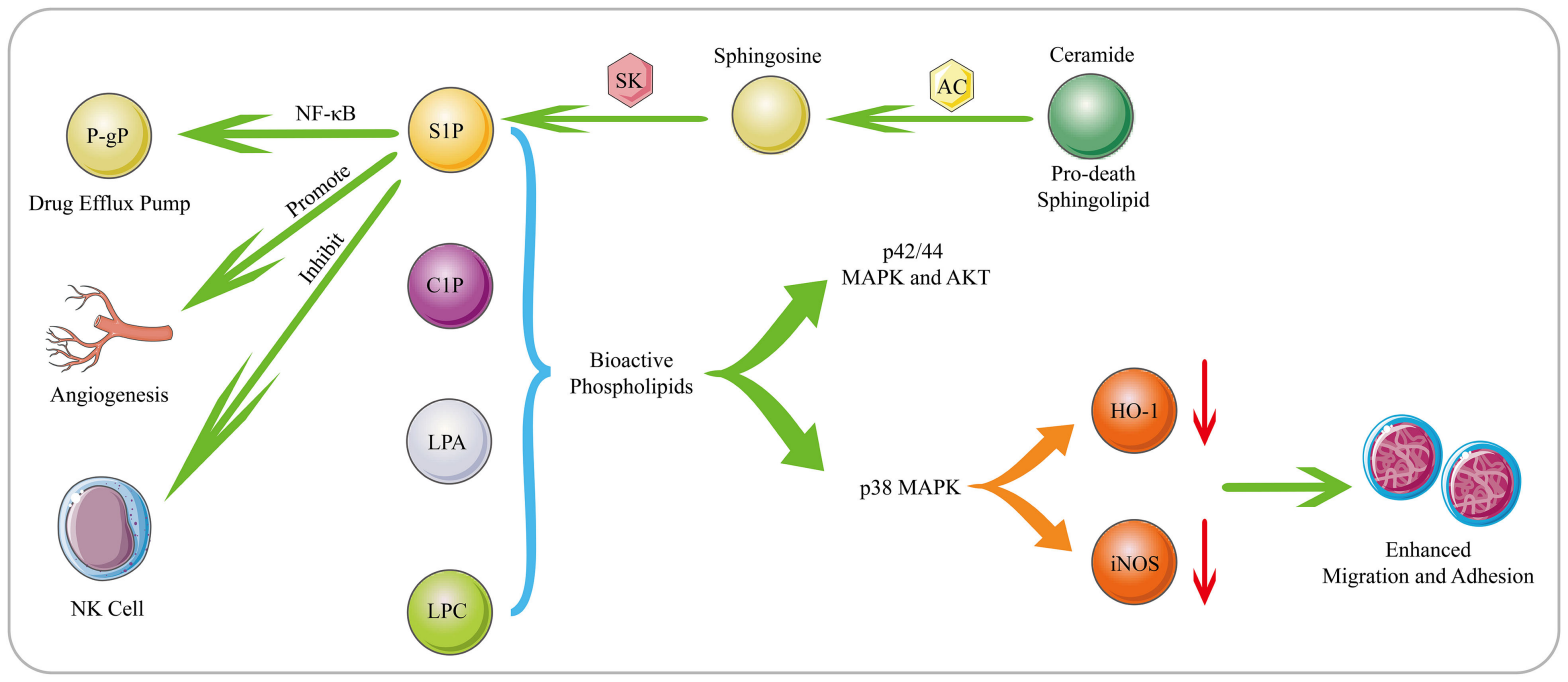
Biological Functions of Bioactive Phospholipids. S1P is generated through phosphorylation of sphingosine by SK. S1P mediates tumor cell proliferation, invasion, angiogenesis, drug resistance and immune escape. Moreover, AC can decompose the pro-death sphingolipid ceramide to generate sphingosine, which subsequently generates S1P. AC and S1P can activate the NF-κB pathway, and mediating the expression of the drug efflux pump P-gp. The bioactive phospholipids such as S1P, C1P, LPC and LPA can stimulate the p42/44 MAPK and AKT signaling pathways. Moreover, as substances that can inhibit the migration of hematological cells, HO-1 and iNOS can be down-regulated by bioactive phospholipids in a p38 MAPK-dependent manner, thereby promoting the migration and adhesion of human leukemia cells.

Phospholipids, as a large class of lipids, are the main components of biological membranes and important signaling molecules for cell proliferation and growth (98). Sphingosine 1-phosphate (S1P), which is generated by phosphorylation of sphingosine by sphingosine kinase 1 (SK1), is an important lipid metabolite that mediates cellular signal transduction (99). There have been numerous reports that S1P mediates tumor cell proliferation, invasion, angiogenesis, and anti-apoptosis (**Figure 2**) (100, 101). In addition, overexpression of SK1 also induces malignant transformation and promotes tumor proliferation (102). Michael S. Lee (103) found that S1P is up-regulated in MCL, and S1P can inhibit the activation of NK cells and allow MCL cells to escape immune immunity. Once targeting S1P or inhibiting SK1, the killing effect of NK cells can be restored on MCL cells, and accompanied by the up-regulation of cardiolipin, phosphatidylcholine, phosphatidylethanolamine and sphingomyelin, especially the up-regulation of cardiolipin suggests that cardiolipin induce the activation of NK cells. Furthermore, S1P has also been reported to interact with S1PR3 to promote osteosarcoma proliferation, anti-apoptosis and aerobic glycolysis through the YAP/c-MYC/PGAM1 axis (104).

Phospholipids also play an important role in the occurrence and development of tumor cells and the generation of drug resistance. SK1 is overexpressed in DLBCL, and its downstream product S1P can induce angiogenesis and promote cell proliferation and growth (105). For the increased expression of SK2 in large granular lymphocytic leukemia, inhibiting the expression of SK2 can lead to the degradation of the downstream pro-survival protein Mcl-1, and ultimately induce cell apoptosis (106). In addition to S1P, bioactive phospholipids such as ceramide 1-phosphate (C1P), lysophosphatidylcholine (LPC) and LPA also promote tumor progression. These bioactive phospholipids can stimulate the p42/44 MAPK and AKT signaling pathways. In addition, as substances that can inhibit the migration of hematological cells, HO-1 and iNOS can be down-regulated by bioactive phospholipids in a p38 MAPK-dependent manner, thereby promoting the migration and adhesion of human leukemia cells (107). Lysophospholipase D enzyme converts lysophospholipids into more water-soluble LPA, which activates GPCR-mediated signaling pathways and produces important lipid mediators. They are required to maintain chronic myelogenous leukaemia stem cells function *in vivo* (108). In MM patients, acid sphingomyelinase (ASM) is significantly up-regulated, which can lead to the increase of ceramide and the decrease of sphingomyelin to cause phospholipid metabolism disorders. The exosomes secreted by MM contain a large amount of ASM, which can make recipient cells resistant to melphalan and BZ (109). Overexpressed Acid ceramidase (AC) in AML can decompose pro-death sphingolipid ceramide to generate sphingosine and FFA, which are converted to S1P by SK1. AC and S1P together stimulate the activation of the NF-κB pathway, which in turn causes the expression of the ATP-binding cassette transporter P-gp. P-gp mediates the excretion of multiple drugs, ultimately conferring resistance to chemotherapeutics in AML (**Figure 2**) (110).

## Conclusion

The rapid growth and continuous invasion of tumor cells require a large amount of energy supply, and metabolic reprogramming is commonly used to meet the material and energy requirements of tumor cells. As an important component of various biological membranes, lipids are also important substances involved in energy storage, production and cell signaling, and play an important role in cell physiological activities (111). Therefore, lipid metabolism reprogramming can be used to meet the material and energy required for rapid proliferation and growth of tumor cells, and lipid metabolism reprogramming has become one of the new markers of cancer (112). In this review, we summarize the proliferation, growth, differentiation, migration, invasion, apoptosis, drug resistance, immune escape and oncogenic mechanisms of tumor cells due to the lipid metabolism reprogramming in hematological malignancies, including FAs, cholesterol, and phospholipids. Tumor cells can increase lipid metabolism by enhancing endogenous lipid *de novo* synthesis pathway and exogenous lipid uptake, including the overexpression of FASN, ACC1, HMGCR, CD36, FABP and LDLR. Moreover, SREBPs have specific important roles in regulating lipid homeostasis, and SREBPs have three subtypes: SREBP-1a, SREBP-1c and SREBP-2. SREBP-1c mainly regulates the expression of genes required for FA synthesis, while SREBP-1a can regulate FA and cholesterol synthesis, as well as cholesterol uptake. SREBP-2 is relatively specific for the regulation of cholesterol synthesis and uptake (113). SREBPs need to be escorted by SCAP from the ER to the Golgi apparatus to perform their biological functions (114).

Lipid metabolism reprogramming plays an important role in the physiological activities of tumor cells, so targeting the lipid metabolism pathway of tumor cells has become an effective therapeutic approach. For example, inhibition of key rate-limiting enzymes in lipid biosynthesis reduces lipid synthesis, overstimulation of lipid biosynthesis causes ER stress, disruption of mitochondrial oxidative homeostasis causes mitochondrial stress, and blocking lipid-related signaling pathways causes signal pathway dysregulation. However, targeting lipid metabolism reprogramming still faces many challenges for the treatment of hematological malignancies. The main reason is that the relevant mechanisms of lipid metabolism are not fully revealed in hematological malignancies. Therefore, lipid synthesis, storage, utilization and efflux cannot be effectively regulated in hematological malignancies. Ferroptosis, which is a hot research topic in recent years, is also closely related to lipid metabolism. Ferroptosis is a programmed cell death caused by excessive accumulation of iron-dependent lipid peroxidation and reactive oxygen species, and various hematological malignancies are sensitive to ferroptosis. Therefore, ferroptosis is also a promising therapeutic strategy for hematological malignancies. However, what we need to pay attention to is that glucose metabolism, lipid metabolism and amino acid metabolism are interconverted and affect each other, which is a complex connection. These results in that single inhibition of a certain metabolism cannot effectively inhibit the growth of tumor cells because the salvage of other metabolisms is activated. Tumor cells acquire the function of MYC through chromosomal translocations, gene amplifications and single nucleotide polymorphisms, causing a variety of metabolic dysregulations. For example, transporters and enzymes of glycolysis, fatty acid synthesis, glutaminolysis, serine metabolism and mitochondrial metabolism (115). The single inhibition of a downstream metabolic change is not effective in inhibiting cancer development. Therefore, combined inhibition of lipid metabolism, glucose metabolism and amino acid metabolism needs to be considered in clinical applications. In conclusion, understanding the oncogenic mechanism of lipid metabolism and targeting lipid metabolism reprogramming to find new therapeutic targets has important scientific significance and clinical application value. This review provides experience and direction for targeting lipid metabolism reprogramming in the treatment of hematological malignancies.

## Author contributions

Conception and design: LZ, LS, and WC. Initial manuscript writing: LZ, NC, LS, and WC. Revision of the manuscript: JL, NC, ZL, and YW. Confirmation of Manuscript: LS and WC. All authors contributed to the article and approved the submitted version.

## Funding

This review was funded by the National Natural Science Foundation of China (No. 82141113; No. 81901511).

## Acknowledgments

We thank all those who participated in the writing of this article. At the same time, we are very grateful to those who have provided us with excellent advice and encouraged us when we faced challenges.

## Conflict of interest

The authors declare that the research was conducted in the absence of any commercial or financial relationships that could be construed as a potential conflict of interest.

## Publisher’s note

All claims expressed in this article are solely those of the authors and do not necessarily represent those of their affiliated organizations, or those of the publisher, the editors and the reviewers. Any product that may be evaluated in this article, or claim that may be made by its manufacturer, is not guaranteed or endorsed by the publisher.
